# Genetic variation and population structure of clonal *Zingiber zerumbet* at a fine geographic scale: a comparison with two closely related selfing and outcrossing *Zingiber* species

**DOI:** 10.1186/s12862-021-01853-2

**Published:** 2021-06-09

**Authors:** Rong Huang, Yu Wang, Kuan Li, Ying-Qiang Wang

**Affiliations:** 1 Guangzhou Key Laboratory of Subtropical Biodiversity and Biomonitoring, School of Life Sciences, South China Normal University, Guangzhou, 510631 China; 2 Guangdong Provincial Key Laboratory of Biotechnology for Plant Development, School of Life Sciences, South China Normal University, Guangzhou, 510631 China

**Keywords:** Reproduction mode, Genetic differentiation, Spatial genetic structure, *Zingiber zerumbet*

## Abstract

**Background:**

There has always been controversy over whether clonal plants have lower genetic diversity than plants that reproduce sexually. These conflicts could be attributed to the fact that few studies have taken into account the mating system of sexually reproducing plants and their phylogenetic distance. Moreover, most clonal plants in these previous studies regularly produce sexual progeny. Here, we describe a study examining the levels of genetic diversity and differentiation within and between local populations of fully clonal *Zingiber zerumbet* at a microgeographical scale and compare the results with data for the closely related selfing *Z. corallinum* and outcrossing *Z. nudicarpum*. Such studies could disentangle the phylogenetic and sexually reproducing effect on genetic variation of clonal plants, and thus contribute to an improved understanding in the clonally reproducing effects on genetic diversity and population structure.

**Results:**

The results revealed that the level of local population genetic diversity of clonal *Z. zerumbet* was comparable to that of outcrossing *Z. nudicarpum* and significantly higher than that of selfing *Z. corallinum*. However, the level of microgeographic genetic diversity of clonal *Z. zerumbet* is comparable to that of selfing *Z. corallinum* and even slightly higher than that of outcrossing *Z. nudicarpum*. The genetic differentiation among local populations of clonal *Z. zerumbet* was significantly lower than that of selfing *Z. corallinum*, but higher than that of outcrossing *Z. nudicarpum*. A stronger spatial genetic structure appeared within local populations of *Z. zerumbet* compared with selfing *Z. corallinum* and outcrossing *Z. nudicarpum*.

**Conclusions:**

Our study shows that fully clonal plants are able not only to maintain a high level of within-population genetic diversity like outcrossing plants, but can also maintain a high level of microgeographic genetic diversity like selfing plant species, probably due to the accumulation of somatic mutations and absence of a capacity for sexual reproduction. We suggest that conservation strategies for the genetic diversity of clonal and selfing plant species should be focused on the protection of all habitat types, especially fragments within ecosystems, while maintenance of large populations is a key to enhance the genetic diversity of outcrossing species.

**Supplementary Information:**

The online version contains supplementary material available at 10.1186/s12862-021-01853-2.

## Background

For most vascular plant species, there are two distinct modes of reproduction: sexual and asexual, through vegetative reproduction and apomixis [1, 2]. For plants reproducing asexually, vegetative reproduction is extremely common in perennial plants [1, 3]. The two modes of reproduction are accompanied by contrasting genetic consequences [2, 4, 5]. Understanding the effects on genetic diversity of the means of reproduction used by plants could provide significant insights into their evolutionary biology and conservation, and thus this has attracted extensive concern and research interest [6].

Plants that employ sexual reproduction can achieve gene exchange between individuals and populations via pollen and seed, while plants relying solely on vegetative reproduction are generally not readily dispersed far from the parent plant [4, 7–9]. Thus, for plants reproducing sexually, there is opportunity for the addition of new genotypes via gene flow and/or genetic recombination [10]. For plants reproducing asexually, in contrast, it is predicted that genetic variation will reduce as a result of the absence of segregation and genetic recombination [11–13], as evidenced by various studies on genetic variation in clonal plants [14, 15]. In contrast to the theoretical predictions, many empirical studies on genetic variation of clonal plants, however, have shown that their population genetic diversity, on average, is generally similar to, or even higher than that of sexually reproducing plants [11, 16–20]. This has led to the widely accepted idea that average genetic variation in populations of clonal plants generally appears as diverse as that of non-clonal plants [18, 21, 22]. In fact, the maintenance of within-population genetic diversity of clonally reproducing species should take into account several facts, e.g. the recruitment of new genotypes through sexually produced diaspores, diversifying selection in different environmental conditions and somatic mutation [11, 16, 17]. The most common reason behind a high level of genetic diversity in populations of many clonal plants with a capacity for sexual reproduction is the sporadic and limited episodes of sexual reproduction [13, 18, 23–30]. For example, asexually reproducing populations of the shrub *Acacia carneorum* contained multiple genets, which can be attributed to occasional sexual recruitment [13], and perennial clonal herbaceous *Maianthemum bifolium* showed high genotypic diversity resulting from very limited sexual recruitment [18]. However, these previous studies did not take into account the mating system of the non-clonal plants concerned, which is the most important factor affecting genetic diversity and spatial genetic structure of populations within species [31–33]; that is, a comparison was not made with selfing and outcrossing plants. For example, Meloni et al. [34] did not consider the breeding system when comparing the genetic diversity of clonal species and sexually reproducing species in the Canary Islands. Moreover, it is worth noting that most clonal plants in these previous studies regularly produce sexual progeny. Numerous studies on genetic variation have shown that, compared to outcrossing plants, self-fertilizing plants have less genetic diversity at both the population and species levels, but genetic differentiation among populations is strengthened [35, 36]. Therefore, given the influences of mating system on genetic diversity, it can be difficult to fully understand the impact of clonal reproduction on genetic variation and spatial genetic structure of populations of clonal plants regularly producing sexual progeny, because its roots lie in a complex mixture of factors. Undoubtedly, studies comparing obligatory clonal plants with selfing and outcrossing plants are needed and could contribute to an improved understanding of the effect of clonal reproduction on population genetic diversity and spatial genetic structure of plants. In addition, the value of previous studies for analysis of clonal reproduction effects on genetic diversity is reduced by lack of consideration of phylogeny. Phylogenetic relationships between the species in focus may confound the comparative analyses of genetic diversity between unrelated asexual and sexual plant species [31, 37], as phylogenetically related species may exhibit combinations of character values that are inherited to some degree from a common ancestor [38]. Given this, comparative studies of closely related taxa have the advantage of being able to better isolate the effects of variation in single traits (e. g. modes of reproduction) on genetic structure [31]. Therefore, comparative studies on population genetic structure between closely related obligatory clonal plant species and sexual plant species can disentangle the phylogenetic effect on genetic variation.

In this study, we focus on a fully clonal plant species from China, *Zingiber zerumbet* (L.) Smith, which is a perennial herb species of section Zingiber of the genus *Zingiber*, closely related to selfing *Z. corallinum* and outcrossing *Z. nudicarpum* (Additional file 1: Fig. S1) [39, 40]. All of the three *Zingiber* plants are highly ornamental and can be used medicinally [39–42]. Our previous studies comparing the genetic structure of the two closely related selfing and outcrossing *Zingiber* species at landscape level [43] and fine scale level [44] have shown that selfing *Z*. *corallinum* can maintain a high level of genetic diversity at both geographic scales, similar to that of outcrossing *Z*. *nudicarpum*, albeit with low genetic diversity within populations or subpopulations. Here, therefore, we assess the levels of genetic variation and differentiation within and among local populations of fully clonal *Zingiber zerumbet* at a microgeographic scale using ISSR markers, and compare the results with those for the two closely related species, selfing *Z*. *corallinum* and outcrossing *Z*. *nudicarpum.* The genetic variation and structure of plant populations can reveal useful information about, and is regarded as the strategic mainstay of, biodiversity and the diversity of a species within and among wild populations inhabiting an ecosystem [45, 46]. Thus, an increased understanding of genetic diversity and genetic structure in species found in habitats is key to the development of conservation strategies for small and isolated populations [47, 48]. In this study, we focus on the following questions. (1) Is the genetic diversity lower in clonal *Zingiber* species than in selfing and outcrossing *Zingiber*, as theory predicts? (2) Are there differences in the spatial genetic structure among *Zingiber* populations at microgeographic scales related to mode of reproduction?

## Results

### ISSR polymorphism, genetic diversity and clonal diversity

The ISSR polymorphism, genetic diversity and clonal diversity data are summarized in Tables 1 and 2. The primers produced 293 reliable ISSR bands from four local populations of *Z*. *zerumbet* across Dongshui Mountain, of which 250 (85.32%) were polymorphic, there were 38 specific bands (12.97%). At the microgeographic level, the values of Nei’s gene diversity (*h*) and Shannon’s genetic diversity index (*I*) were 0.2409 and 0.3713, respectively. At the local population level, the values of *h* and *I* ranged from 0.1140 to 0.1971 (average 0.1448) and from 0.1695 to 0.2955 (average 0.2157), respectively. The 12 selected primers identified 205 genotypes from 229 individuals, and 199 (86.9%) of those were unique. The number of genotypes (*G*) per local population ranged from 24 at MLH to 105 at HJC (average 51.3), and the number of unique genotypes ranged from 24 at MLH and DS1 to 104 at HJC (average 49.8). Simpson’s diversity index (*D*) was 1.00 for all individuals at microgeographic level, and ranged from 0.90 to 1.00 (average 0.97) at local population level.

**Table 1:** Attributes of ISSR primers of *Zingiber zerumbet* used in the present study

Primer	Sequence 5´ to 3´	*T*m (°C)	SR (bp)	NT	NP
810	(GA)8 T	44	220–2350	22	13
811	(GA)8 T	52	230–2050	22	20
817	(CA)8A	56	270–1750	21	20
826	(AC)8C	56	230–1800	27	21
834	(AG)8Y*T	46	320–2100	23	23
841	(GA)8Y*C	56	180–1850	24	24
847	(CA)8R^*^C	59	180–2200	38	35
857	(AC)8Y*G	48.5	180–1950	24	19
884	HBH*(AG)7	52.5	220–1850	23	20
887	DVD*(TC)7	52	370–1950	22	20
888	BDB*(CA)7	56	270–2050	21	13
889	DBD*(AC)7	59	270–1850	26	22
Total	–	–	180–2350	293	250

*B = (C, G, T), D = (A, G, T), R = (A, T), V = (A, C, G), Y = (C, G), H = (A, C, T)

*T*m annealing temperature, *SR* size range of amplified fragments, *NT* total number of bands, *NP* number of polymorphic bands

**Table 2:** Comparison of genetic diversity and clonal diversity parameters based on ISSR for local populations of *Zingiber zerumbet* at the microgeographic scale with that of selfing *Z. corallinum* and outcrossing *Z. nudicarpum*. (The data of *Z. corallinum* and *Z. nudicarpum* utilized in this study are from Huang et al. [44].)

Local population	Sample size	*G*	*S*	*G*/*N*	*D*	*PL*	*PPL* (%)	*N*a	*N*e	*h*	*I*	NS
*Z. zerumbet*												
DS1	45	26	24	0.58	0.90	95	32.42	1.3242	1.1972	0.1140	0.1695	7
DS2	54	50	47	0.93	0.99	116	39.59	1.3959	1.2258	0.1342	0.2024	5
MLH	24	24	24	1.00	1.00	102	34.81	1.3481	1.2402	0.1340	0.1955	5
HJC	106	105	104	0.99	1.00	174	59.39	1.5653	1.3399	0.1971	0.2955	21
Average	57	51.3	49.8	0.90	0.97	122	41.55	1.4084	1.2508	0.1448	0.2157	9.5
Total	229	205	199	0.90	1.00	250	85.32	1.8532	1.3952	0.2409	0.3713	38
*Z. corallinum*												
Average	33					38	20.30	1.2028	1.1129	0.0662	0.0995	11.3
Total	115					157	84.20	1.7655	1.4245	0.2490	0.3753	39.5
*Z. nudicarpum*												
Average	34					119	52.60	1.5256	1.2421	0.1464	0.2257	5
Total	86					194	83.80	1.8384	1.3768	0.2246	0.3480	12.5

*G* number of genotypes, *S* number of genotypes found only once, *G*/*N* the number of genotypes (G) relative to that of samples (N), *D* Simpson’s diversity index, *PL* number of polymorphic loci, *PPL* percentage of polymorphic loci, *N*a number of observed alleles, *N*e number of effective alleles, *h* Nei's gene diversity, *I* Shannon’s information index, *NS* number of specific bands

**Fig. 1:**
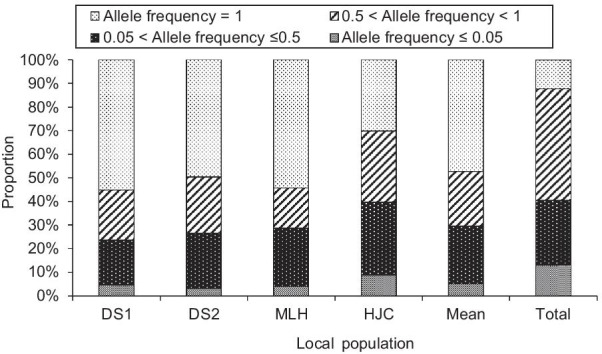
Distribution of allele frequency in local populations of *Zingiber zerumbet* within the microgeographic area

The patterns of allele frequency are shown in Fig. 1. Among all local populations except HJC, common loci (i.e. found in all individuals within local populations: allele frequency = 100%) accounted for the highest proportion (49.78%–55.19%) of amplified fragments, followed by low-medium allele frequency loci (5% < allele frequency ≤ 50%) (18.87%–24.66%) and medium–high allele frequency loci (50% < allele frequency < 100%) (17.04%–23.81%). However, common loci, medium–high allele frequency loci and low-medium allele frequency loci accounted for a similar proportion of amplified fragments in HJC, i.e. 30.12% and 30.12% and 30.92%, respectively. Rare loci (allele frequency ≤ 5%) accounted for the lowest proportion (3.14%–8.84%) of amplified fragments in all four subpopulations. At the microgeographic level, both common loci (i.e. found in all local populations within the microgeographic area: allele frequency = 100%) and rare loci were less prevalent, i.e. 12.32% and 13.03%, respectively. However, medium–high allele frequency loci accounted for the highest proportion (47.18%) of amplified fragments, followed by the low-medium allele frequency loci (27.46%).

### Genetic differentiation and gene flow at the microgeographic scale

The genetic differentiation statistics for all subpopulations are presented in Table 3. The Nei’s *G*_ST_ values for the local populations of *Z*. *zerumbet* at the microgeographic scale were estimated as 0.4008, which indicates that 59.92% of the genetic variability was distributed within local populations. The estimate of gene flow (*N*m) per generation among the local populations was 0.7476. The AMOVA analysis was consistent with the Nei’s genetic differentiation statistics, showing that 46.0% (*Φ*_ST_ = 0.460) of the total variation was partitioned among local populations of *Z*. *zerumbet* at the microgeographic scale (Table 4). That is, of the total molecular variance, 54.0% was attributable to individual differences in *Z*. *zerumbet*.

**Table 3:** Comparison of genetic differentiation statistics between local populations of clonal *Zingiber zerumbet* at the microgeographic scale with that of selfing *Z. corallinum* and outcrossing *Z. nudicarpum*. (The data of *Z. corallinum* and *Z. nudicarpum* utilized in this study are from Huang et al. [44].)

Local populations	*H*_T_	*H*_S_	*G*_ST_	*N*m
*Z. zerumbet*				
DS1 vs MLH	0.1815	0.1242	0.3158	1.0831
DS1 vs DS2	0.1892	0.1243	0.3427	0.9588
DS1 vs HJC	0.2312	0.1557	0.3264	1.0319
MLH vs DS2	0.1701	0.1341	0.2117	1.8623
MLH vs HJC	0.2178	0.1655	0.2401	1.5823
DS2 vs HJC	0.2197	0.1657	0.2459	1.5337
Average	0.2016	0.1449	0.2804	1.3420
Total	0.2299	0.1405	0.4008	0.7476
*Z. corallinum*				
Average	0.1920	0.0622	0.6782	0.3060
Total	0.2484	0.0721	0.7110	0.2575
*Z. nudicarpum*				
Average	0.2013	0.1449	0.2680	1.6596
Total	0.2269	0.1474	0.3408	1.0311

*H*_T_ total microgeographic diversity, *H*_S_ average within local population diversity, *G*_ST_ local population differentiation, *N*m gene flow

**Table 4:** Comparison of summary of analysis of molecular variance (AMOVA) for local populations of clonal *Zingiber zerumbet* at the microgeographic scale with that of selfing *Z. corallinum* and outcrossing *Z. nudicarpum*. (The data of *Z. corallinum* and *Z. nudicarpum* utilized in this study are from Huang et al. [44])

Subpopulation	Source	df	Sums of squares	Mean squares	Variance component	Percentage of variation (%)	*Ф*_ST_	*p*
*Z. zerumbet*								
	Between local populations	3	2647.505	882.502	16.613	46.0	0.460	0.001
	Within local populations	225	4387.936	19.502	19.502	54.0		
*Z. corallinum*								
	Between local populations	6			23.942	78.4	0.784	0.001
	Within local populations	224			12.152	21.6		
*Z. nudicarpum*								
	Between local populations	4			19.451	46.8	0.468	0.001
	Within local populations	168			17.172	53.2		

*df* degrees of freedom, *Ф*_ST_ between local populations deviations from Hardy–Weinberg expectations, *p* the probability of accepting the null hypothesis

### Genetic structure and cluster analysis at the microgeographic scale

**Fig. 2:**
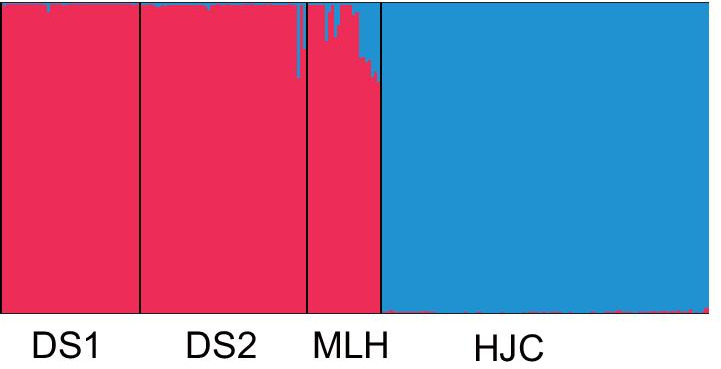
Genetic-group-structure shown by STRUCTURE analysis for local populations of *Zingiber zerumbet* within the microgeographic area. Each individual vertical bar represents an individual and the black vertical bars separate the local populations, while different colors represent different gene pools

Bayesian genetic analyses performed with STRUCTURE revealed that with the log likelihood reached its maximum value at K = 2, when all individuals from four local populations of *Z*. *zerumbet* could be assigned to two genetic clusters (Fig. 2). Except for population MLH, almost all individuals within each local population were assigned to the same genetic clusters. The local populations DS1, DS2 and MLH were assigned to the same cluster and HJC was assigned to a second cluster. In MLH, there was a high degree of admixing of two gene pools.

**Fig. 3:**
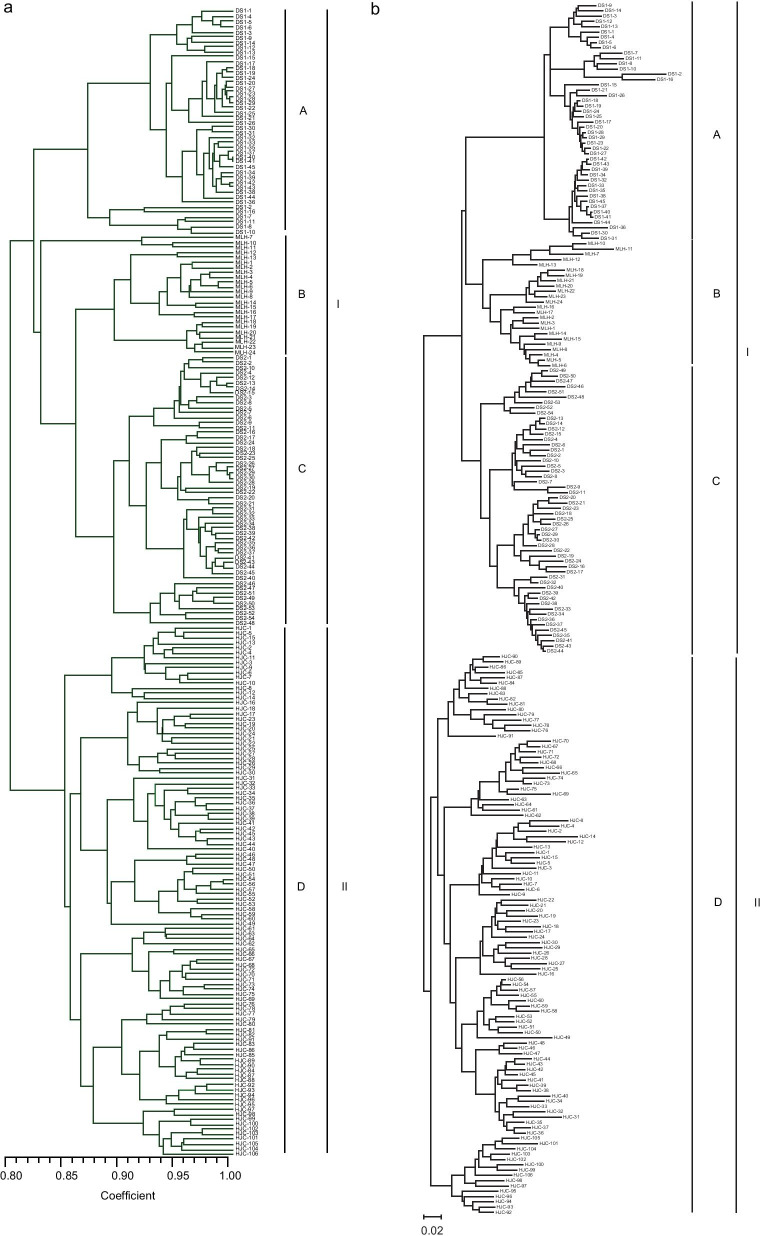
UPGMA dendrogram **a** based on the Dice coefficient and an unrooted Neighbor-Joining tree, **b** based on Nei’s genetic distance for individuals in local populations of *Zingiber zerumbet* within the microgeographic area

**Fig. 4:**
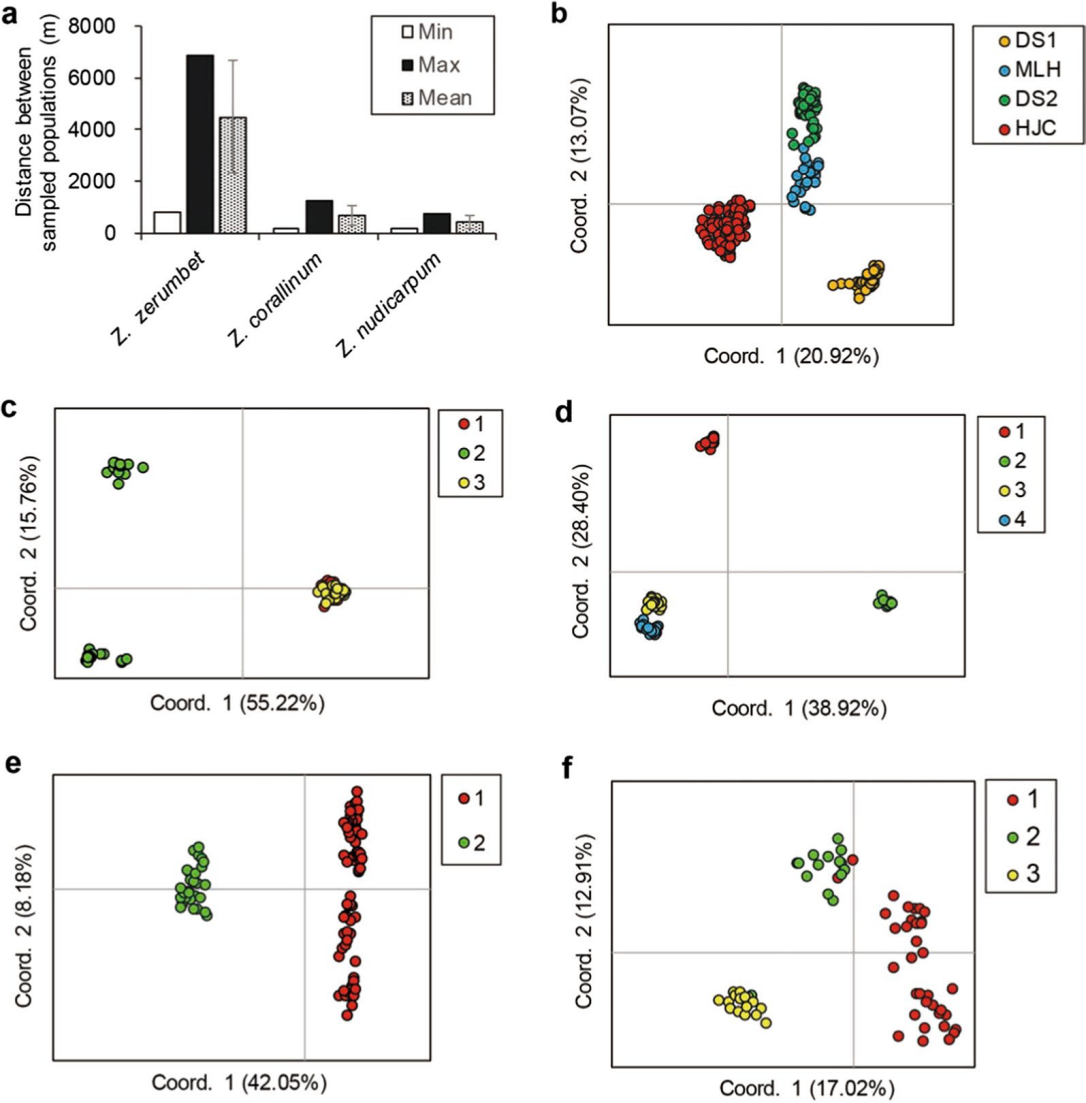
Comparison of scatterplot of the principal coordinate analysis (PCoA) based on ISSR polymorphisms for individuals in local populations of clonal *Zingiber zerumbet* within the microgeographic area with that of selfing *Z. corallinum* and outcrossing *Z. nudicarpum*. Different colors represent individuals from different local populations. (**a** minimum, maximum, and mean distance between sampled populations of the three *Zingiber* species, **b** *Z*. *zerumbet*, **c**, **d** *Z. corallinum*, **e**, **f** *Z. nudicarpum*; the figures of *Z. corallinum* and *Z. nudicarpum* utilized in this study are from Huang et al. [44])

**Fig. 5:**
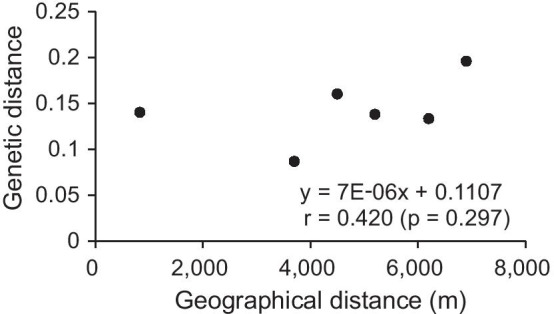
Correlation between geographic distance and Nei’s genetic distance between local populations of *Zingiber zerumbet* within the microgeographic area

The UPGMA dendrogram (Fig. 3a) based on the Dice coefficient was broadly consistent with the unrooted neighbor-joining (NJ) tree (Fig. 3b) based on Nei’s genetic distance in local populations of *Z*. *zerumbet* at the microgeographic scale. All individuals from the same local populations were clustered together with the exception of three individuals from MLH when using an arithmetic average analysis. The 229 individuals were first grouped into two clusters (I, II) and then cluster I formed three further well-resolved clades (A, B and C) comprising all individuals from local populations DS1, MLH and DS2, respectively (Fig. 3). Cluster II consisted of clade D only, which comprised all individuals from HJC. Except for seven individuals, all the neighboring individuals within local populations clustered together (Fig. 3a, b). The PCoA broadly confirmed the partitioning results of the UPGMA and NJ clustering (Fig. 4b). The Mantel Test revealed that there was no significant isolation-by-distance relationship across local populations of *Z*. *zerumbet* at the microgeographic scale (r = 0.420, *p* = 0.297) (Fig. 5).

**Fig. 6:**
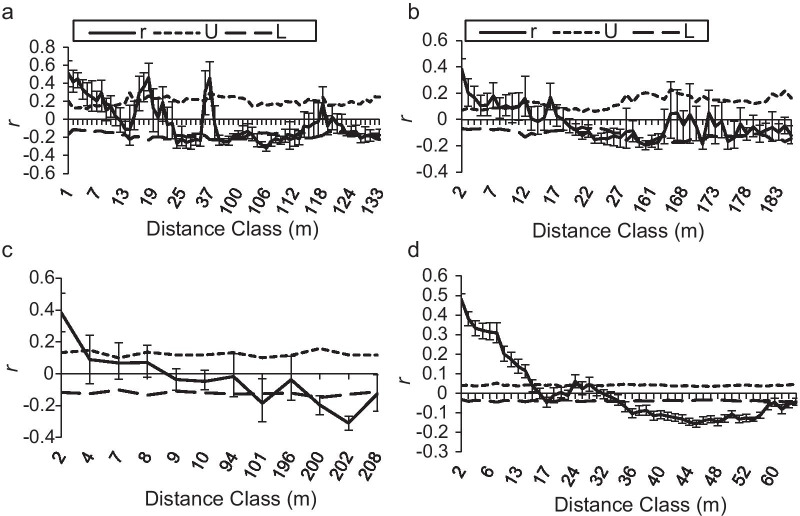
Correlogram showing the spatial autocorrelation coefficient (*r*) within four local populations of *Zingiber zerumbet* within the microgeographic area. (U and L represent the 95% two-tailed confidence interval, which was calculated based on 999 permutations; a-d–local populations DS1, DS2, MLH and HJC)

The spatial autocorrelation analysis indicated that significant positive spatial genetic structure was detected at 2–14 m (*r* = 0.268 ± 0.125, *p* < 0.05) within local populations of *Z*. *zerumbet* (Fig. 6).

## Discussion

### Is the genetic diversity of a clonal *Zingiber* species lower or higher than that of sexual *Zingiber* species?

Genetic uniformity could be expected within populations of strictly clonal species [25]. Contrary to theoretical predictions, some comparative studies on genetic variation between unrelated clonal and sexual plants species have shown that the genetic variation of the former is comparable to that of the latter [18, 21, 22]. Nevertheless, there are a few studies comparing genetic diversity in populations between closely related clonal plants and non-clonal or less clonal ones (e. g. *Typha angustifolia* and *T*. *latifolia* [49]; *Gagea lutea* and *G*. *spathacea* [50]), showing lower levels of genetic diversity in populations of clonal plants. The present study revealed that the level of local population genetic diversity in clonal *Z*. *zerumbe*t was comparable to that in closely related outcrossing *Z*. *nudicarpum* (*h*: 0.1448 vs 0.1464, *p* = 0.95; *I*: 0.2157 vs 0.2257, *p* = 0.79) and significantly higher than that in closely related selfing *Z*. *corallinum* (*h*: 0.1448 vs 0.0662, *p* = 0.05; *I*: 0.2157 vs 0.0995, *p* = 0.05). However, the level of microgeographic genetic diversity of clonal *Z*. *zerumbet* is comparable to that of selfing *Z*. *corallinum* (*h*: 0.2409 vs 0.2490, *p* = 0.587; *I*: 0.3713 vs 0.3753, *p* = 0.838), and even slightly higher than that of outcrossing *Z*. *nudicarpum* (*h*: 0.2409 vs 0.2246, *p* = 0.389; *I*: 0.3713 vs 0.3480, *p* = 0.493). These results suggest that, compared with outcrossing *Z*. *nudicarpum*, the genetic diversity of local populations of clonal *Z. zerumbet* at the microgeographic scale will be less affected by reduced gene flow because each individual contains most of the genetic variation within the population, similar to selfing *Z*. *corallinum* [44]. In addition, the clonal diversity of *Z*. *zerumbet* at the microgeographic scale was also relatively high, with most of the sampled plants representing unique genotypes (199 genets out of 229 sampled). The observed values of clonal diversity of *Z*. *zerumbet* (*G*/*N* = 0.90; *D* = 0.97) were higher than the average values of clonal plant species in several literature surveys (e.g. *G*/*N* = 0.17, *D* = 0.67 for 21 clonal species summarized by Ellstrand and Roose [16]; *G*/*N* = 0.27; *D* = 0.75 for 45 clonal species reported by Widén et al. [17]; *G*/*N* = 0.44; *D* = 0.85 for 77 clonal species reported by Honnay and Jacquemyn [19]). This may be because these previous reviews of clonal diversity included taxa with various levels of sexual reproduction. It indicates that obligatory clonal plant species may have higher clonal diversity than other clonal species with various levels of sexual reproduction. We suggest that fully clonal plant species (such as *Z*. *zerumbet*) are able not only to maintain a high level of within-population genetic diversity as outcrossing plant species (such as *Z*. *nudicarpum*), but can also harbor as high a level of microgeographic genetic diversity as selfing plant species (such as *Z*. *corallinum*), albeit by adopting diverse strategies.

Unlike outcrossing plant species, obligatory clonal plant species, like selfing ones, cannot maintain high genetic diversity within/among populations through frequent exchange of genes [51]. However, without migration among demes of local populations (subpopulations) at microgeographic scales (metapopulations) of fully clonal or selfing plant species, any mutation that arises in local populations (subpopulations) may be fixed and cannot spread to other local populations (subpopulations). This is confirmed by the lowest proportion of common loci and the higher proportion of low-medium loci at the microgeographic level in clonal *Z*. *zerumbet* (12.32% vs 27.46%) and selfing *Z*. *corallinum* (6.8% vs 43.2%) [44]. Compared to selfing plant species, obligatory clonal plant species should become more heterozygous for a particular allele in an individual small population fragment because of the absence of a capacity for sexual reproduction, thus resulting in a higher heterozygosity. Furthermore, independent ramets can prove advantageous to reduce the likelihood of the death of a genet, and consequently protecting against loss of genetic variation [3, 17]. This was confirmed by our results, in which the proportion of common loci within local populations of clonal *Z. zerumbet* was marginally lower than that of selfing *Z. corallinum* (47.34% vs 67.62%, *p* = 0.079) and comparable to that of outcrossing *Z*. *nudicarpum* (47.34% vs 37.73%, *p* = 0.187). Moreover, 53.33%–100% of the sampled individuals within local populations of *Z*. *zerumbet* were unique, which may suggest that individuals have diverged with regard to genotypic composition. This may be attributed to random genetic mutations resulting from single-base changes or transposon activation, leading to the accumulation of somaclonal variation in clonal propagation [20, 34, 37, 52, 53]. Thus, the fully clonal plant *Z. zerumbet* may increase genetic variation within populations by the accumulation of random somatic mutations, as found in some cloned plants [3, 26, 34, 54]. Numerous studies have also shown that per-generation mutation rates in plants that reproduce vegetatively are generally expected to be higher than non-clonal plants as result of a growing number of somatic mutations [2, 55, 56]. In addition, clonal plants can be specialized for varying environmental conditions [10, 56, 57]. Habitats with contrasting environments have long been known to promote the co-existence of locally adapted genotypes through diversifying selection [17, 57, 58], thus resulting in high levels of genetic diversity [17, 27]. In this case, ecological differences between local populations at the microgeographic scale may also have contributed to the maintenance of genetic diversity of *Z*. *zerumbet*. The population-specific bands detected in every local population of *Z*. *zerumbet* at the microgeographic scale also imply that local populations may diverge due to different environmental conditions.

Based on the above, we suggest that conservation strategies for the genetic diversity of clonal plants, like those for selfing species, should be focused on the protection of all habitat types, especially isolated fragments within ecosystems, while maintenance of large populations is a key to enhancing genetic diversity for outcrossing species.

### Are the differences in the population genetic structure of *Zingiber* species at microgeographic scales related to mode of reproduction?

Compared to outcrossing species, fully clonal plants, like selfing plants, theoretically have lower genetic diversity within populations and higher differentiation between populations [59, 60], since pollen migration between populations is rare or absent in clonal and selfing plants, and a specific locus arising in an individual population cannot spread to other populations [35, 61]. However, the genetic differentiation between local populations (subpopulations) of clonal *Z*. *zerumbet* was significantly lower than that of selfing *Z*. *corallinum* (*G*st = 0.4008 vs 0.7110, *p* = 0.00) and higher than that of outcrossing *Z*. *nudicarpum* (*G*st = 0.4008 vs 0.3408, *p* = 0.486) in this study. AMOVA analysis also showed that more variation was found within local populations of clonal *Z*. *zerumbet* (54.0%) and outcrossing *Z*. *nudicarpum* (53.2%) at microgeographic scales, but the opposite was true for selfing *Z*. *corallinum* (21.6%). Unlike outcrossing *Z*. *nudicarpum*, clonal *Z*. *zerumbet* obviously cannot counter genetic differentiation between local populations through gene flow [62], due to absence of migration of pollen and seeds within /among local populations at the microgeographic scale. However, prolonged clonal growth may buffer the effect of genetic drift, leading to reducing genetic differentiation between populations, as a result of the persistence of clonal propagation [9, 18]. This is the case for clonal *Z*. *zerumbet* in our study. Furthermore, there is no inbreeding depression that could lead to increasing genetic differentiation among populations in fully clonal plants. Thus, we suggest that the fully clonal plants may have higher genetic diversity within populations and lower differentiation among populations compared to selfing plants, similar to that of outcrossing plants, albeit as a result of different strategies.

Compared with outcrossing species, selfing species always tend to show a stronger spatial genetic structure, due to lack of gene flow via pollen [63, 64], which is evidenced by the result of comparison between selfing *Z. corallinum* and outcrossing *Z. nudicarpum* [44]. High levels of clonal clustering are always present in plants that reproduce by vegetative propagation, due to limited dispersal ability [2, 8, 65, 66], which is confirmed by the results of our cluster analysis, showing that neighboring individuals within local populations of clonal *Z*. *zerumbet* always cluster together at the microgeographic scale, similar to selfing *Z. corallinum* (Additional file 1: Figs. S2, S3) [44]. However, many individuals do not aggregate with their neighbors within subpopulations (local populations) in outcrossing *Z. nudicarpum* (Additional file 1: Figs. S4, S5) [44]. Moreover, there is an absence of migration of pollen and seeds within /among local populations of fully clonal plants. Thus, fully clonal plants should generate a stronger spatial genetic structure than plants employing sexual reproduction [2, 65]. Our result is consistent with this hypothesis, as evidenced by the significant positive spatial genetic structure at a smaller spatial scale (2–14 m) compared with selfing *Z*. *corallinum* (2–34 m) and outcrossing *Z*. *nudicarpum* (500–1500 m) [44]. For clonal *Z*. *zerumbet*, this is the logical consequence, because the dispersal distances of vegetative propagules in *Z*. *zerumbet* are expected to be shorter than those of pollen and seed in sefling *Z*. *corallinum* and especially in outcrossing *Z*. *nudicarpum*.

## Conclusions

In the present study, our results revealed that, contrary to theoretical predictions, fully clonal *Zingiber zerumbet* is able not only to maintain as high a level of within-population genetic diversity as outcrossing *Z. nudicarpum*, but can also harbor as high a level of microgeographic genetic diversity as selfing *Z. corallinum*, probably due to the accumulation of somatic mutations and absence of a capacity for sexual reproduction. There are differences in the spatial genetic structure of *Zingiber* populations at microgeographic scales and these are related to mode of reproduction. The present study advances our understanding of the effect of the means of reproduction used by plants on population genetic diversity and genetic structure.

## Methods

### Study species, study sites and sample collection

**Fig. 7:**
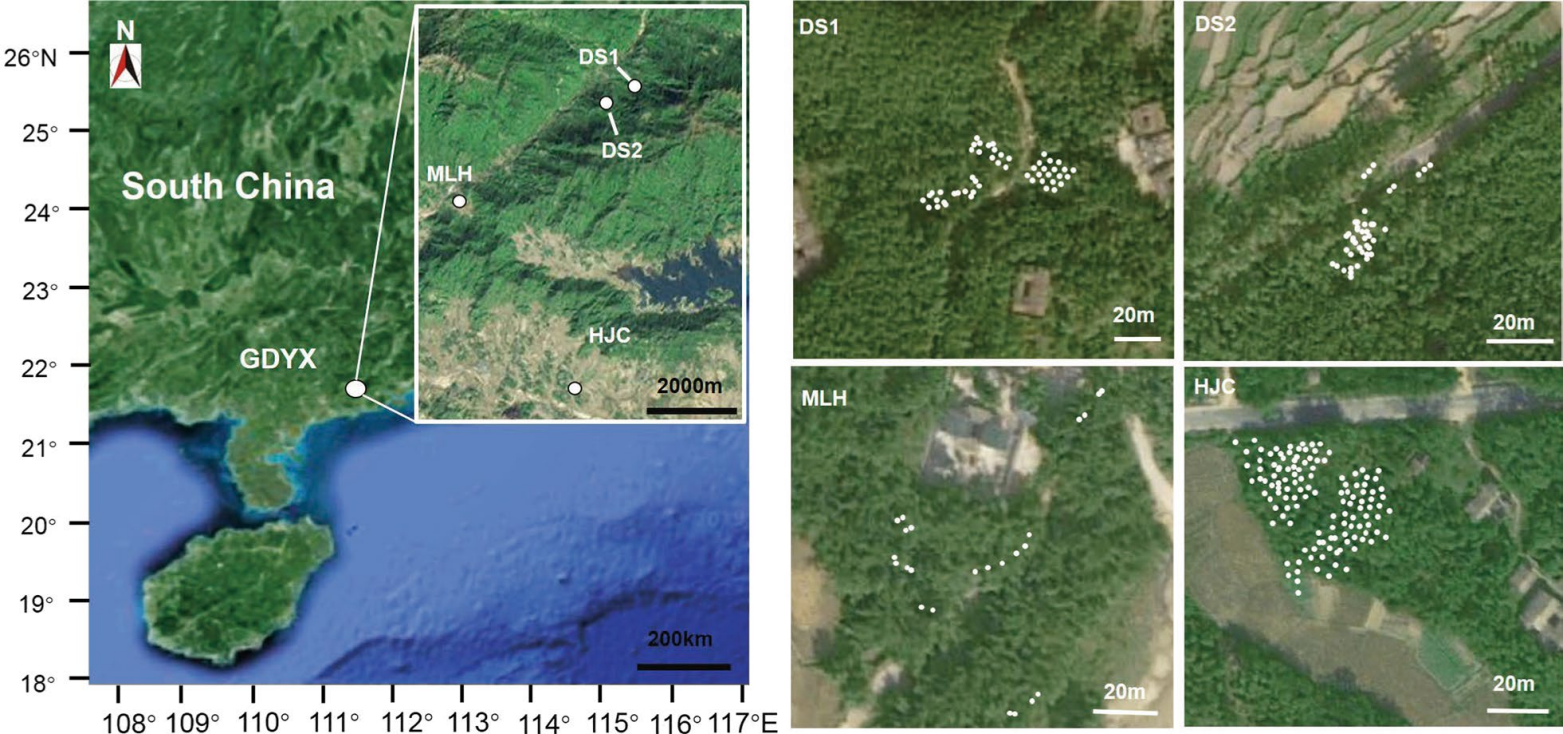
Location of the microgeographic area (GDYX) and distribution of local populations of *Zingiber zerumbet* within GDYX. Each dot represents a sampled individual. The map was drawn by the authors with reference to Google Maps. The map can be found at https://maps.google.com/

*Zingiber zerumbet* (L.) Smith is diploid perennial herb with hermaphrodite flowers, like selfing *Z*. *corallinum* and outcrossing *Z*. *nudicarpum*, and is widespread in moist places in forests, distributed across tropical regions such as south China, Cambodia, India, Laos, Malaysia, Myanmar, Sri Lanka, Thailand and Vietnam [39, 40]. However, during our 15 years (2005–2020) of fieldwork, no fruiting has been observed in any natural populations of *Z*. *zerumbet* in China, and thus it is considered to be sterile, reproducing vegetatively through rhizome elongation [67]. This can be confirmed by the absence of seed set after hand pollination [41, 42]. In this study, we sampled all local populations within an area of ca. 3 × 6 km^2^ across Dongshui Mountain (village) in Yangxi County (GDYX—21° 47′ 28″ N, 111° 25′ 43″ E, alt. 215–272 m) (Fig. 7), Guangdong Province, China, in order to examine the microgeographic genetic variation and genetic structure of clonal *Z. zerumbet*. The species has spatially structured populations with four local populations in different habitats naturally isolated by ca. 830–6900 m (average 4500 m) of agricultural land, village, mountain forest, or stream. The individuals grow in open bamboo forest land near a village (HJC), on abandoned farmland (DS1) and margins of remnant forest (DS2) and alongside a stream (MLH).

In order to analyze within-population spatial genetic structure, we collected samples from 24 to 106 individuals throughout the full spatial extent of each local population (Table 2). Spatial distances between neighboring samples that appeared unconnected to each other were at least 2 m to minimize the risk of resampling the same clone. The straight-line distance between individuals was also estimated directly on the basis of the site coordinates to calculate the spatial autocorrelation coefficient (*r*) within local populations. Leaf tissue samples were stored in silica gel prior to DNA analysis. Voucher specimen was collected (voucher number: wyq-14-45) for *Z. zerumbet* and deposited at the herbarium of South China Normal University (SN). The species was identified by professor Ying-Qiang Wang from School of Life Sciences, South China Normal University. Permissions were not necessary for collecting these samples, because they do not grow in nature reserves or included in the list of national key protected plants. Our field works and molecular experiments complied with local legislation, national and international guidelines. We also abide by the Convention on the Trade in Endangered Species of Wild Fauna and Flora.

### DNA extraction and PCR

Total genomic DNA from the sampled leaves was extracted using a modified CTAB method [68]. The quality and quantity of DNA were assessed using 0.8% agarose gel electrophoresis and a spectrophotometer. ISSR-PCR amplifications were performed on a BIO-RAD T100 thermal cycler with an initial denaturation for 5 min at 95 °C, followed by 39 cycles of denaturation for 45 s at 94 °C, annealing for 45 s and extension for 90 s at 72 °C, with a final extension for 10 min at 72 °C. Ten primers that have been used previously in *Z*. *corallinum* and *Z*. *nudicarpum* [43, 44] were used for *Z*. *zerumbet*, together with an additional two primers for this species (Table 1). PCR was carried out in a total volume of 20 μL, including 40 ng template DNA, 2.0 μL 10× buffer, 1.50 mmol Mg^2+^, 0.15 mmol dNTPs, 0.8 μmol primer, 2.0 units of Taq DNA polymerase and double-distilled water. To test for possible contamination, negative controls, in which template DNA was replaced with distilled water, were included in each PCR set. The amplification products were subjected to electrophoresis on 1.8% agarose gels in 0.5 × TBE buffer at 130 V for 1–1.5 h along with a 100 bp ladder, and photographed using a gel documentation system (Bio-Rad GelDoc XR^+^). To ensure repeatability of the results, duplicate PCR amplifications were performed and only clear and reproducible bands were scored and used in the final analysis. The images of the gels were analyzed using Image Lab software (Bio-Rad) to score for the presence (1) or absence (0) of bands and to assign a fragment size to each band using an algorithm based on the 100 bp ladder. The presence or absence of bands was further confirmed by eye. A binary matrix of the ISSR phenotypes was built based on the presence or absence of bands. The binary matrix of the ISSR phenotypes was stored in the figShare repository (https://doi.org/10.6084/m9.figshare.14593941).

### Data analysis

#### Analysis of genetic diversity, clonal diversity, differentiation and gene flow

The binary matrix was used to calculate the genetic diversity parameters at local population and microgeographic level using POPGENE v. 1.32 [69]. These parameters were as follows: percentage of polymorphic loci (*PPL*), observed number of alleles (*N*a), effective number of alleles (*N*e), Nei’s gene diversity (*h*), and Shannon’s information index (*I*). Samples with similarities above 0.98 were considered to be identical and to belong to the same genotype [70]. Two measures of clonal diversity for each local population were used in our study as follows: (1) The “proportion distinguishable” (*G*/*N* ratio), which was defined as the number of genotypes (*G*) divided by the number of samples (*N*) [16]; (2) Simpson’s diversity index (*D*), which was defined as 1-∑[*n*_*i*_(*n*_*i*_ − 1)/N(N − 1)], where *n*_*i*_ is the number of samples of genotype *i* and N is the total number of samples collected for that local population [71]. *G*_ST_, an indicator of the degree of differentiation between local populations, was calculated using POPGENE. To assess the values of population genetic differentiation (*Φ*) and the proportion of total variation among and within local populations, the matrix was also subjected to an analysis of molecular variance (AMOVA) implemented in GenAlEx 6.502 [72] based on 999 permutations. Gene flow (*N*m) between local populations was calculated as *N*m = 0.5(1 − *G*_ST_)/*G*_ST_ [73].

#### Analysis of genetic structure

STRUCTURE v. 2.1 [74] was used to derive the number of genetic units and to assign an individual to K genetic clusters. We performed five runs for each value of k, with a run length of 1,000,000 Markov chain Monte Carlo (MCMC) replications after a burn-in period of 100,000. In order to illustrate the genetic relationship between individuals, a dendrogram was constructed using an unweighted paired group method with an arithmetic average analysis (UPGMA) based on the Dice coefficient in NTSYSpc-2.10 [75]. A neighbor-joining (NJ) tree was also generated in MEGA v. 7 [76] from the genetic distances matrix [77]. Principal Coordinates Analysis (PCoA) using GenAlEx provided a visual representation of the genetic relationships within and between local populations of *Z*. *zerumbet*. Mantel tests implemented in GenAlEx were performed to examine whether genetic distances between local populations were related to the corresponding measures of geographic distance.

#### Spatial genetic structure (SGS) within local populations

To investigate genetic relatedness of individuals with respect to spatial position within local populations, single population spatial structure analyses were performed to assess the spatial genetic structure of the studied local populations using GenAlEx. To reduce noisy confidence limits in case of uneven sampling, even sample classes were chosen [72]. The autocorrelation coefficient (*r*) calculated according to Smouse and Peakall [78] is similar to Moran’s-*I*, ranging from -1 to 1. To test the statistical significance of the spatial autocorrelation values, a classic two-tailed 95% confidence interval (CI) was generated and bootstrap resampling was performed 999 times.

## Supplementary Information

**Additional file 1: Fig S1–S5**.** Fig. S1**. Inflorescence of* Zingiber zerumbet*,* Z. nudicarpum* and* Z. corallinum*.** Fig. S2**. UPGMA dendrogram based on Dice coefficient for individuals in metapopulations of* Zingiber corallinum* (A–GDZJ, B–GDYX). The figure utilized in this study is from Huang et al. [44].** Fig. S3**. Unrooted Neighbor-Joining trees based on Nei’s genetic distance for individuals in metapopulations of* Zingiber corallinum* (A–GDZJ, B–GDYX). The figure utilized in this study is from Huang et al. [44].** Fig. S4**. UPGMA dendrogram based on Dice coefficient for individuals in metapopulations of* Zingiber nudicarpum* (A–HNCJ, B–HNBT). The figure utilized in this study is from Huang et al. [44].** Fig. S5**. Unrooted Neighbor-Joining trees based on Nei’s genetic distance for individuals in metapopulations of* Zingiber nudicarpum* (A–HNCJ, B–HNBT). The figure utilized in this study is from Huang et al. [44].

## Data Availability

The binary matrix of the ISSR phenotypes is available in the figShare repository (https://doi.org/10.6084/m9.figshare.14593941). The map in Fig. 7 is available on https://maps.google.com/.

## Abbreviations

*G*: Number of genotypes
*S*: Number of genotypes found only once
*G*/*N*: The number of genotypes (G) relative to that of samples (N)
*D*: Simpson’s diversity index
*PL*: Number of polymorphic loci
*PPL*: Percentage of polymorphic loci
*N*a: Number of observed alleles
*N*e: Number of effective alleles
*h*: Nei's gene diversity
*I*: Shannon’s information index
NS: Number of specific bands
*T*m: Annealing temperature
SR: Size range of amplified fragments
NT: Total number of bands
NP: Number of polymorphic bands
*H*_T_: Total microgeographic diversity
*H*_S_: Average within local population diversity
*G*_ST_: Local population differentiation
*N*m: Gene flow
df: Degrees of freedom
*Ф*_ST_: Between local populations deviations from Hardy–Weinberg expectations
*p*: The probability of accepting the null hypothesis
