# Supplementary material for: Genetic variation and population structure of clonal Zingiber zerumbet at a fine geographic scale: a comparison with two closely related selfing and outcrossing Zingiber species
Source: BMC Ecol Evol. 2021 Jun 9;21:116. doi: 10.1186/s12862-021-01853-2 (PMC8191059; doi:10.1186/s12862-021-01853-2)
Supplement: Supplementary file 1 — Additional file 1: Fig S1–S5. Fig. S1. Inflorescence of Zingiber zerumbet, Z. nudicarpum and Z. corallinum. Fig. S2. UPGMA dendrogram based on Dice coefficient for individuals in metapopulations of Zingiber corallinum (A–GDZJ, B–GDYX). The figure utilized in this study is from Huang et al. [44]. Fig. S3. Unrooted Neighbor-Joining trees based on Nei’s genetic distance for individuals in metapopulations of Zingiber corallinum (A–GDZJ, B–GDYX). The figure utilized in this study is from Huang et al. [44]. Fig. S4. UPGMA dendrogram based on Dice coefficient for individuals in metapopulations of Zingiber nudicarpum (A–HNCJ, B–HNBT). The figure utilized in this study is from Huang et al. [44]. Fig. S5. Unrooted Neighbor-Joining trees based on Nei’s genetic distance for individuals in metapopulations of Zingiber nudicarpum (A–HNCJ, B–HNBT). The figure utilized in this study is from Huang et al. [44]. [file 12862_2021_1853_MOESM1_ESM.docx]

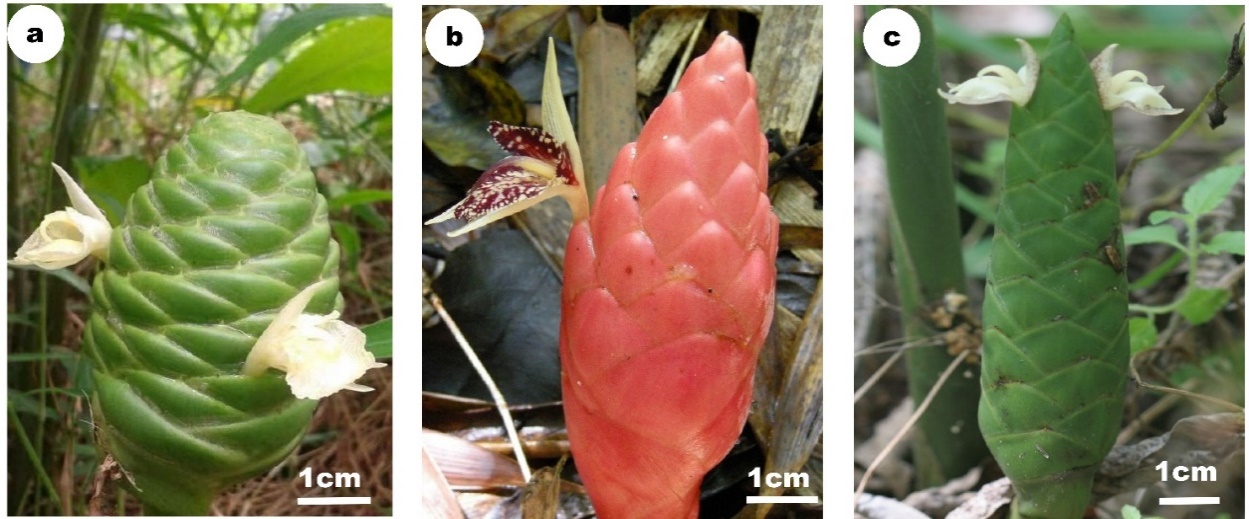
**Fig. S1** Inflorescence of *Zingiber zerumbet* (a), *Z. nudicarpum* (b) and *Z. corallinum* (c).


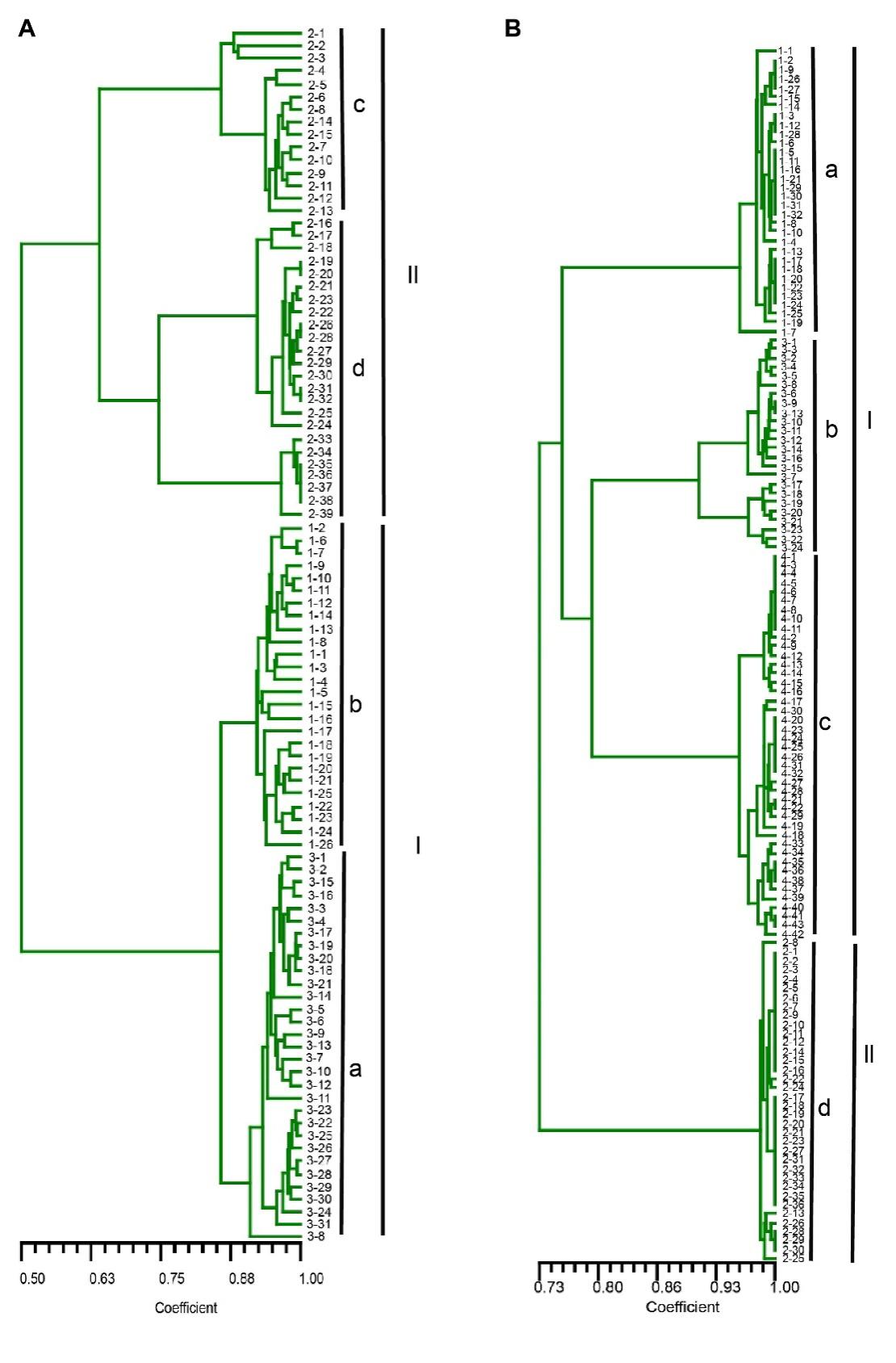
**Fig. S2** UPGMA dendrogram based on Dice coefficient for individuals in metapopulations of *Zingiber corallinum* (A--GDZJ, B--GDYX). The figure utilized in this study is from Huang et al [44].

**Fig. S**
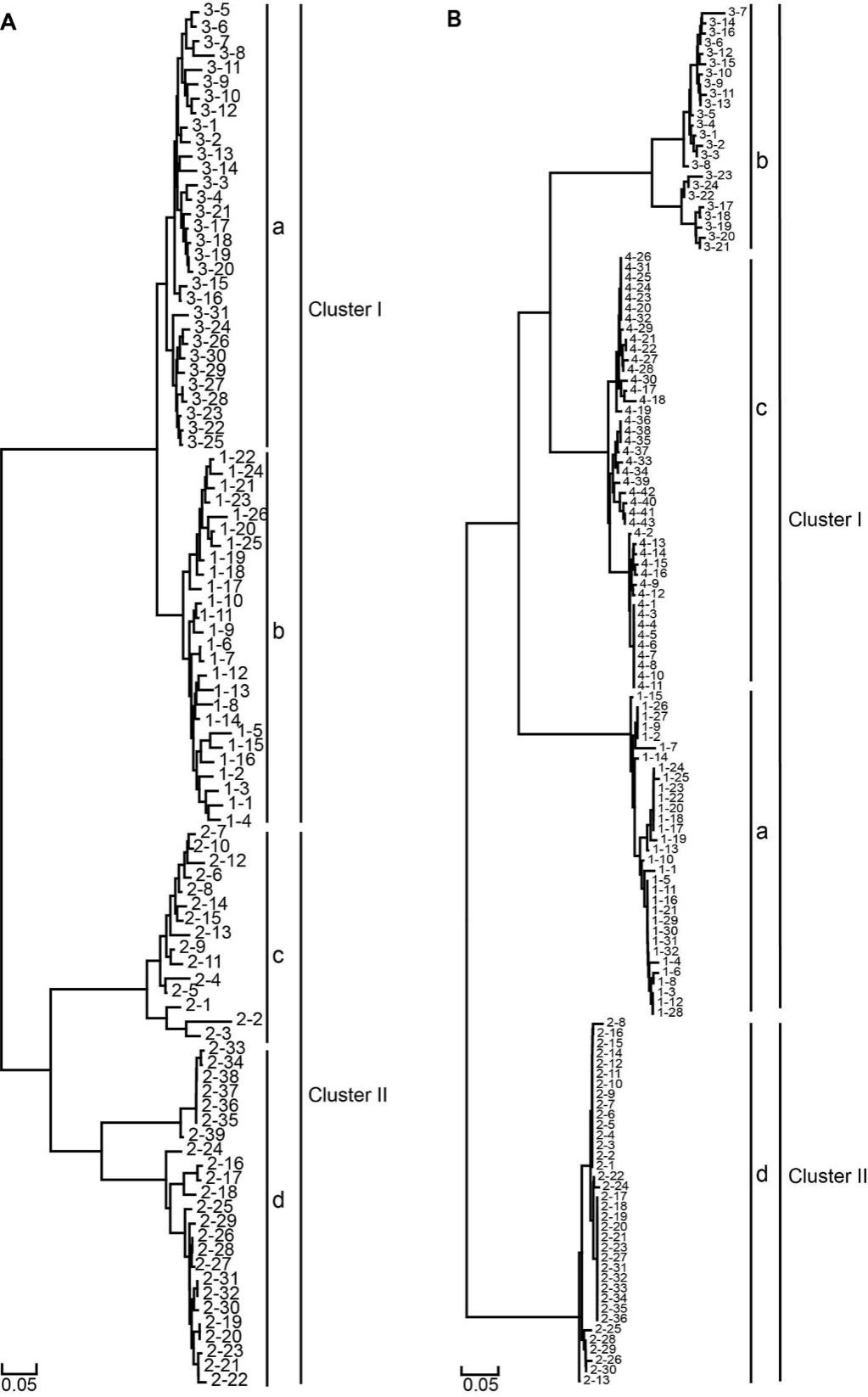
**3** Unrooted Neighbor-Joining trees based on Nei’s genetic distance for individuals in metapopulations of *Zingiber corallinum* (A--GDZJ, B--GDYX). The figure utilized in this study is from Huang et al [44].

**
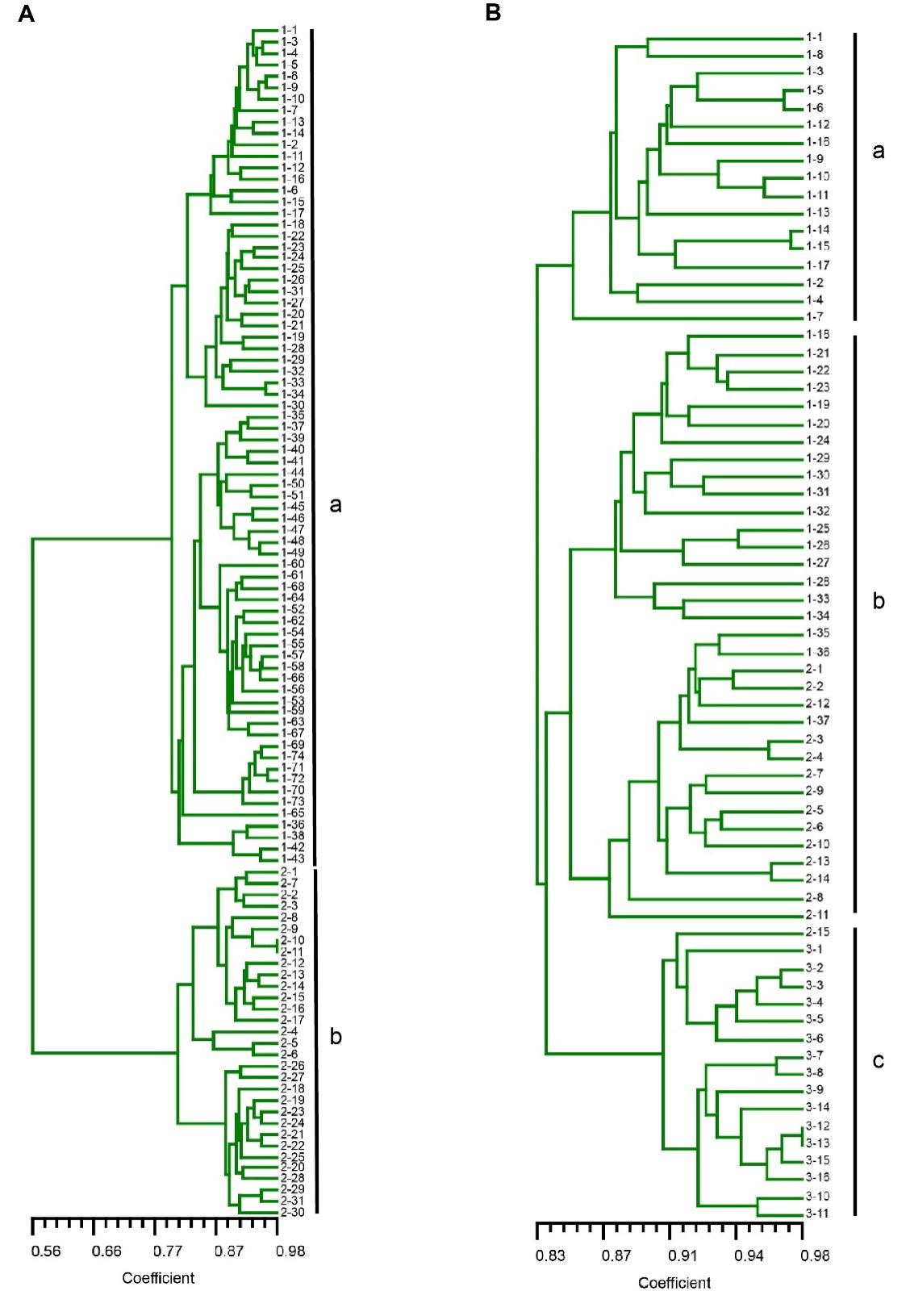
Fig. S4** UPGMA dendrogram based on Dice coefficient for individuals in metapopulations of *Zingiber nudicarpum* (A--HNCJ, B--HNBT). The figure utilized in this study is from Huang et al [44].

**Fig. S**
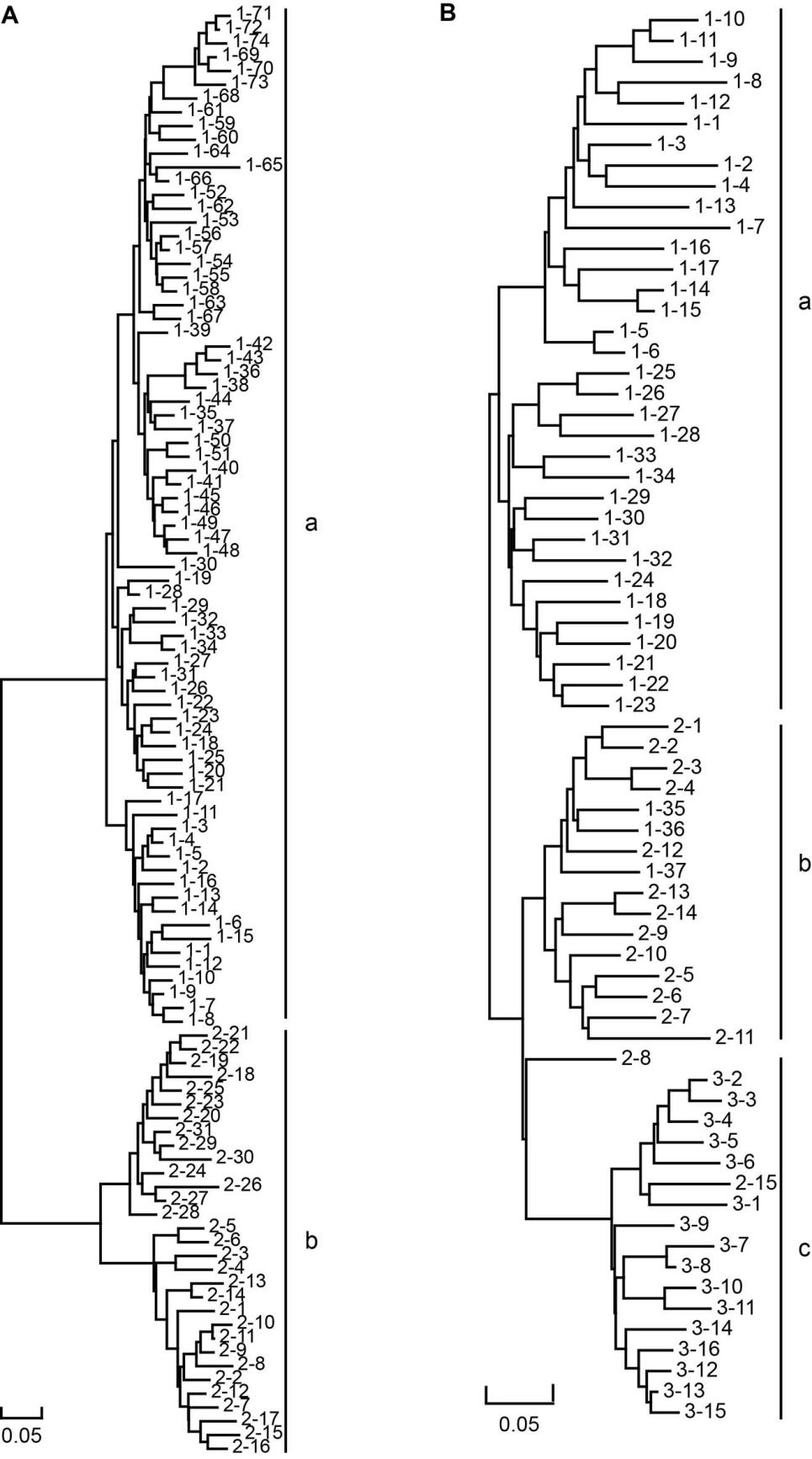
**5** Unrooted Neighbor-Joining trees based on Nei’s genetic distance for individuals in metapopulations of *Zingiber nudicarpum* (A--HNCJ, B--HNBT). The figure utilized in this study is from Huang et al [44].
